# Anticancer Molecular Mechanisms of Epigallocatechin Gallate: An Updated Review on Clinical Trials

**DOI:** 10.1002/fsn3.70735

**Published:** 2025-08-01

**Authors:** Ahmad Mujtaba Noman, Muhammad Tauseef Sultan, Aimen Mazhar, Iqra Baig, Jawaria Javaid, Muzzamal Hussain, Muhammad Imran, Suliman A. Alsagaby, Waleed Al Abdulmonem, Ahmed Mujtaba, Tadesse Fenta Yehuala, Mohammed M. Ghoneim, Ehab M. Mostafa, Mohamed A. Abdelgawad

**Affiliations:** ^1^ Department of Human Nutrition, Faculty of Food Science and Nutrition Bahauddin Zakariya University Multan Pakistan; ^2^ TIMES Institute Multan Multan Pakistan; ^3^ Department of Nutrition and Health Promotion University of Home Economics Lahore Pakistan; ^4^ Department of Food Science Government College University Faisalabad Faisalabad Pakistan; ^5^ Department of Food Science and Technology University of Narowal Narowal Pakistan; ^6^ Department of Medical Laboratory Sciences, College of Applied Medical Sciences Majmaah University AL‐Majmaah Saudi Arabia; ^7^ Department of Pathology, College of Medicine Qassim University Buraidah Saudi Arabia; ^8^ Department of Food Science and Technology, Faculty of Engineering Sciences and Technology Hamdard University Islamabad Campus Islamabad Pakistan; ^9^ Faculty of Chemical and Food Engineering, Bahir Dar Institute of Technology Bahir Dar University Bahir Dar City Ethiopia; ^10^ Department of Pharmacy Practice, College of Pharmacy AlMaarefa University Ad Diriyah Riyadh Saudi Arabia; ^11^ Department of Pharmacognosy, College of Pharmacy Jouf University Sakaka Saudi Arabia; ^12^ Pharmacognosy and Medicinal Plants Department, Faculty of Pharmacy (Boys) Al‐Azhar University Cairo Egypt; ^13^ Department of Pharmaceutical Chemistry, College of Pharmacy Jouf University Sakaka Saudi Arabia

**Keywords:** anticancer, antioxidant, EGCG, green tea, IL‐6, NF‐κB, polyphenols, TNF‐α

## Abstract

Human physiology is a complex process encompassing various biochemical mechanisms, regulated by hundreds of chemical mediators, thus making it more complicated to understand. Balance between these mediators is mandatory to continue the helm of life; however, an imbalance can disrupt normal physiological functions and lead to chronic health conditions. Cancer is one such chronic state that occurs due to an imbalance or overproduction of reactive oxygen and nitrogen species (RONS), which are highly unstable and damage other chemical constituents in the body. The mutilation at the cellular level results in DNA and genetic mutation, uncontrolled cell proliferation, metastasis, organ dysfunction, and ultimately mortality. Various types of cancers are intimidating human life globally, and lung cancer is leading them all, followed by breast and colorectal cancer. Oxidative stress (OS) is the main root cause of cancers that occur due to overproduced RONS. Tumor suppressor genes (TSGs) like p53, PTEN, INK4, MADR2, APC, and oncogenes such as HER2, BCR/ABL1, CMYC, EML4AK, RAS, WNT, ERK, and TRK get mutated by OS. Moreover, various pro‐inflammatory markers i.e., IL‐1, IL‐6, TNF‐α, IFN‐γ, TGF‐β and growth factors (VEGF, EGF, IGF‐2) are also involved in cancer progression. Along with these aspects, the oncogenesis involves different signaling pathways/axis such as MAPK, PI3K, mTOR, Wnt/β‐catenin, GSK3, and NF‐κB. Epigallocatechin gallate (EGCG), a bioactive compound abundantly existing in green tea and grapes with strong antioxidant activity and anticancer potential, is a suitable approach to reduce the cancer burden. The in vitro and in vivo anticancer studies of EGCG proved anticancer and anti‐tumor effects through modulation of cancer signaling pathways, reduction in cell proliferation, decreased metastasis, suppressed angiogenesis, enhanced antioxidant activity, inhibited pro‐inflammatory biomarkers, improved TSGs expression, and downregulated oncogenes expression. Shortly, the current review focuses on the anticancer potential of EGCG through possible mechanisms.

## Graphical abstract

Epigallocatechin gallate (EGCG), a bioactive compound abundantly exists in green tea and grapes with strong antioxidant activity and anticancer potential is a suitable approach to reduce the cancer burden. The invitro and invivo anticancer studies of EGCG proved anticancer and anti‐tumor effect through modulation of cancer signaling pathways, reduction in cell proliferation, decreased metastasis, suppressed angiogenesis, enhanced antioxidant activity, inhibited pro‐inflammatory biomarkers, improved TSGs expression and downregulated oncogenes expression. Shortly, the current review focuses on anticancer potential of EGCG through possible mechanisms.

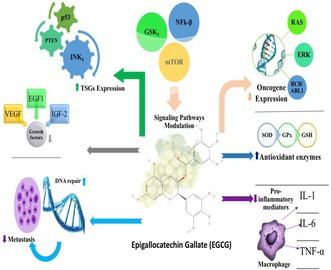

**Abbreviations**
ARs: androgenic regulators
BC: breast cancer
BMPs: bone morphogenic proteins
BPH: benign prostatic hyperplasia
CAFs: cancer‐associated fibroblasts
cagA: cytotoxin‐linked gene A
CC: cervical cancer
CP: chronic pancreatitis
CRC: colorectal cancer
EBV: Epstein–Barr virus
EC: endometrial cancer
EGCG: epigallocatechin gallate
EGCG‐Pip NPs: EGCG‐piperine nanoparticles
EMT: epithelial mesenchymal transition
ER: endoplasmic reticulum
ER: estrogen receptor
FLD: fatty liver disease
GC: gastric cancer
GPI: glycosylphosphatidylinositol
GTE: green tea extract
HCC: hepatocellular carcinoma
HDACIs: histone deacetylase inhibitors
HDACs: histone deacetylases
HIF‐1: hypoxia‐inducible factor‐1
HRT: hormonal replacement therapy
HTN: hypertension
iNOS: inducible nitric oxide synthase
IR: insulin receptor
JNK: Jun NH2‐terminal kinase
LC: lung cancer
LCFA: acylated long chain fatty acid
miRNAs: microRNAs
MMPs: metalloproteinases
NCT: National Clinical trial
NO: nitric oxide
NPs: nanoparticles
NSCLC: non‐small cell lung cancer
OC: osteosarcoma
OS: oxidative stress
OTC: over the counter
PC: prostate cancer
PDC: pancreatic ductal carcinoma
PIM‐1: serine/threonine
PIN: prostatic intraepithelial neoplasia
PP: pancreatic polypeptide
pRB: retinoblastoma protein
PSMA^+^
: prostate‐specific membrane antigen
Rb: retinoblastoma
RCC: renal cell carcinoma
RCSCs: renal cancer stem cells
RONS: reactive oxygen and nitrogen species
ROS: reactive oxygen species
SCLC: small cell lung cancer
S‐NaCl: saturated NaCl
TAC: total antioxidant capacity
TC: trans‐cinnamaldehyde
TKIs: tyrosine kinase inhibitors
TRAIL: TNF‐related apoptosis‐inducing ligand
TSGs: tumor suppressor genes
UC: uterine cancer
uPARs: urokinase‐type plasminogen activator receptors
VBL: vinblastine
VEGF: vascular endothelial growth factor

## 1 Introduction

Plants‐derived bioactive compounds/phytochemicals with significant therapeutic potential have derived the attention of health care professionals, food sectors, pharmaceutics, and cosmetics industries. The phytochemicals are secondary metabolites of plants, which protect them from pathogenic and pest attacks and provide humans with valuable health benefits. The wide array of these bioactive compounds from various plant sources holds distinct inimitable properties according to their root sources (Ketnawa et al. 2022). In recent decades, humans have become more concerned about their health and demanding ready‐to‐use products with health‐promoting and disease‐preventive aspects. Therefore, these bioactive compounds are available in the form of nutraceuticals and functional foods. The growing global market of nutraceuticals and functional foods has been expanding every year and is expected to reach US billion dollars in the coming years (Fernandes et al. 2019). EGCG, an ester of EGC and gallic acid, is one such bioactive compound that abundantly exists in green tea and grapes with prime importance. Besides green tea, some other sources of EGCG with trace quantities are apple skin, plums, onions, and hazelnuts (Kciuk et al. 2023).

In spite of technology advancement in the health care system, human health and well‐being is still a global issue because mankind has been facing various metabolic, pathogenic, congenital, and degenerative ailments due to different factors. Cancer is a chronic widespread health issue causing millions of morbidities and mortalities around the globe. Various types of cancers contribute separately to fatalities, and lung cancer is leading them all. The main root cause involves the production of RONS, causing OS or oxidative damage, thus leading to DNA damage, genetic aberrations, cell proliferation, and tumorigenesis (Jelic et al. 2021). Irregular dietary patterns, pathogens, heavy metals, chemical toxins, and over‐the‐counter (OTC) drugs are such factors that played their notorious role in cancer progression by promoting RONS and OS. However, cancer management through a balanced and well‐nourished dietary approach is a suitable strategy (Zhang, Pan, et al. 2020; Zhang, Yu, et al. 2020; Zhang, Xu, et al. 2020). Plant‐based bioactive compounds, especially EGCG, have been reported with anticancer potential; therefore, consumption of EGCG from natural plant resources can reduce the risk factors and cancer pathogenesis (Aggarwal et al. 2022).

## 2 Bioavailability of EGCG

The chemical structure of compound, source, processing techniques, and consumers' gastrointestinal environment are crucial factors, playing their role in digestion, absorbance, and bioavailability of specific compound. The chemical structure of EGCG contains a four‐ring core with biological activity; in addition, the phenolic hydroxyl group found on the core ring is accountable for the major medicinal activities of EGCG (Dai et al. 2020). Researchers and scientists are trying to enhance the absorption and bioavailability of EGCG. For instance, the methylation of EGCG can increase its solubility and stability. Moreover, the replacement of the hydroxyl group with a lipophilic methoxy group results in improved stability and bioavailability as well (Forester and Lambert 2015). Acylated long‐chain fatty acid (LCFA) in EGCG and the substitution of hydroxyl groups of EGCG with hydrophilic monosaccharides to improve fat and aqueous solubility, antioxidant activity, and availability have also been reported (Wang et al. 2016).

The absorbance and bioavailability of catechins from tea in the small intestine have been evidenced, and upon entering the blood circulation, they undergo degradation, metabolism, and efflux; thus, this results in decreased bioavailability. Some clinical investigations have revealed that ~1.68% of EGCG in human plasma declines to 0.16% of green tea catechins after 6 h post‐ingestion (Cai et al. 2018). An earlier study found that EGCG levels in humans' plasma were 0.57 μM after the oral administration of 3 g of decaffeinated green tea. Additionally, pharmacokinetic investigations of EGCG reported that after oral administration, < 1% of EGCG was noticed in human blood (Chow and Hakim 2011). The ingested tea catechins are mainly metabolized with significant phase II enzymes, like catechol‐O‐methyltransferase, uridine 5‐diphospho‐glucoronosyltransferase, and sulfotransferase. After this, they are absorbed into the small intestine and liver, whereas the remaining enter the colon. Catechins are particularly absorbed through passive diffusion in the epithelium, such as paracellular and transcellular diffusions, because there are no definite receptors present to transport EGCG on the surface of small intestinal epithelial cells (Santhakumar et al. 2018).

Gut microbiota has a direct relation with the metabolism of EGCG, and their substantial key role is not negligible. In vitro and in vivo research have stated that gut microbiota can damage EGCG; vanˈt Slot and Humpf (2009) proved that gut microbiota in pigs completely metabolized EGCG within 4–8 h by using a pig cecum model. Takagaki and Nanjo (2010) stated that EGCG was primarily hydrolyzed to EGC and gallic acid through rat intestinal bacteria. Hence, it is proved that various factors are influencing digestion, absorbance, and bioavailability of EGCG, and these factors are hindering its potential applications against various metabolic disorders. Some studies documented that, in order to increase bioavailability, one should increase the dose of EGCG, but its heavy dose has been found toxic (Murakami 2014). However, nanoparticle‐based delivery systems have proved effective in enhancing EGCG bioavailability. Cano et al. (2019) enhanced the therapeutic potential of EGCG in APPswe/PS1dE9 Alzheimer's disease mice model via dual‐drug loaded EGCG NPs. They concluded that oral administration of NPs resulted in a 5 times increase in EGCG accretion in all key organs, including the liver and brain. EGCG‐based nanoparticles with increased bioavailability and therapeutic potentials are presented in Table 1.

**TABLE 1:** EGCG‐based nanoparticles with increased bioavailability and therapeutic potential.

	NPs/material	Bioavailability/benefit	References
EGCG	Glutamic acid‐functionalized iron oxide NPs	Anti‐inflammatory and anticancer activity	Yang et al. (2022)
	EGCG/AA NPs	5 times	Cano et al. (2019)
	Folic acid‐ nanostructured lipid carriers with EGCG	1.8 times	Granja et al. (2019)
	Curcumin and α‐tocopheryl succinate‐loaded PS–PSyox–NPs	Breast cancer cells (MDA‐MB‐231)	Karahaliloğlu et al. (2018)
	EGCG‐ZnO crystalline NPs	PC‐3 prostate adenocarcinoma cells	Samutprasert et al. (2018)
	EGCG‐piperine nanoparticles (EGCG‐Pip NPs)	Colo205, MCF7, and HeLa cell lines	Dahiya et al. (2018)
	EGCG‐loaded RGD‐ NLC	Apoptotic activity	Hajipour et al. (2018)
	Lipid‐based NPs	2‐fold greater bioavailability in vivo than free EGCG, higher antioxidant potential	Koutelidakis et al. (2017)
	Protein‐based NPs	EGCG NPs showed 9.82‐, 2.04‐, and 1.72‐fold increase in skin than free EGCG	Shetty et al. (2017)
	Carbs‐based NPs	amplified 2.3‐fold, Caco‐2 cells	Hu et al. (2015)

## 3 Antioxidant Potential

The antioxidant activity of EGCG enables it to deliver significant therapeutic potential against different cancers because antioxidant activity is the ability to neutralize RONS, which are highly unstable and lead to OS and cancer development. EGCG exhibits antioxidant activity through three mechanisms: limitation of respiration in the mitochondrial compartment, DNA complex with protective histones, and eradication of excess RONS by the antioxidant enzymes system (Snezhkina et al. 2019). The OS alleviating properties of EGCG are accomplished via inhibiting pro‐oxidant enzymes like NADPH oxidase and antioxidant systems activation, i.e., SOD, catalase, and GSH, and by diminishing nitric oxide (NO) generated metabolites by inducible nitric oxide synthase (iNOS) (Mokra et al. 2022). Moreno‐Vásquez et al. (2021) explored the antioxidant activity of EGCG‐loaded chitosan NPs and reported that EGCG chitosan NPs showed high antioxidant potential in ABTS, DPPH, and FRAP assays. Li et al. (2022) verified that EGCG and ferulic acid‐loaded CS‐NPs showed antioxidant potential, repaired H_2_O_2_‐induced injured cells at a concentration of 200 μg/mL, and promoted tyrosinase inhibition activity at 25 μg/mL. The EGCG antioxidant activity was high in solution and gel form in the DPPH assay compared with sodium ascorbate and ascorbic acid (Dewantari and Setyabudi 2021).

The association between RONS and inflammation has been fully understood, and it has been found that EGCG can significantly reduce inflammatory markers (TNF‐α, IL‐6, IL‐1β), MMPs, COX, NF‐κB and NO. Ahmed et al. (2020) described the antioxidant role of EGCG via attenuating OS and improved SOD and GPx activity in buffalo spermatozoa. Likewise, Gonzalez‐Alfonso et al. (2019) studied the impact of α‐glucosylation on the stability and antioxidant activity of EGCG. They reported that α‐glucosylation improved EGCG pH and thermal stability, increased free radical scavenging, assessed through the ABTS assay, and boosted REDOX activity. The antioxidant capacity of mono‐nd diacylated EGCG was estimated by DPPH, ABTS, FRAP, Fe2+ chelation, β‐carotene bleaching assays, and LDL and DNA oxidation assays. The results suggested that antioxidant activity fluctuated according to the increased chain length of the acyl group and that acylated EGCGs have great potential as lipophilic substitutes to water‐soluble EGCG in lipid‐based mediums (Peng and Shahidi 2022). Xu et al. (2019) studied pH and temperature outcomes on the stability and antioxidant capacity of EGCG in aqueous medium. They found that EGCG stability declined with increasing pH and temperature. Moreover, the total antioxidant capacity (TAC) of samples under different pH values after thermal treatments was found to be non‐significant. EGCG showed antioxidant activity via inhibited Cd2+−induced apoptosis via scavenging ROS in HL‐7702 cells (An et al. 2014).

## 4 Clinical Trials

The clinical trials conducted on several types of cancer throughout the world have demonstrated the significant anticancer potential of EGCG through modulating different cancer pathways, inhibiting oncogenes expression, promoting TSGs activity, and reducing pro‐inflammatory mediators. Samavat et al. (2019) conducted a placebo‐controlled randomized trial with 538 healthy postmenopausal participants to assess the influence of green tea extract (GTE) on hormones and insulin‐like growth factor proteins, which are associated with breast cancer. They concluded multiple results, including reduced LDL, total cholesterol, and increased estradiol concentrations. Obesity is accompanied by a high risk of prostate cancer; considering this, the trial of Kumar et al. (2017) involving 97 subjects proved that green tea catechins (400 mg) were not effective in reducing obesity markers. Garcia et al. (2014) evaluated the effect of GTE in 98 cervical cancer patients with HPV and low‐grade cervical neoplasia and found that GTE is safe, well tolerated, but has a non‐significant impact on cervical cancer. Table 2 showed the anticancer potential of EGCG in different clinical trials worldwide.

**TABLE 2:** Anticancer potential of EGCG in different clinical trials worldwide (Aggarwal et al. 2022).

NCT* No.	Country	Study type	Subjects/participants	Intervention
02891538	USA	Colon cancer	50	EGCG
02580279	China	Breast cancer	68	EGCG
02577393	China	Lung cancer	83	EGCG
00573885	Canada	Lung cancer	53	GTCE
00666562	USA	Bladder cancer	31	GTE
01105338	UK	Prostate cancer	126	Green tea capsules, green tea drink
00303823	USA	Cervical cancer	98	GTCE
02336087	USA	Pancreatic adenocarcinoma	21	Drugs
02029352	Netherlands	Basal cell carcinoma	42	Sinecatechins 10%

Abbreviation: NCT, National Clinical trial.

## 5 Anticancer Perspectives

**FIGURE 1:**
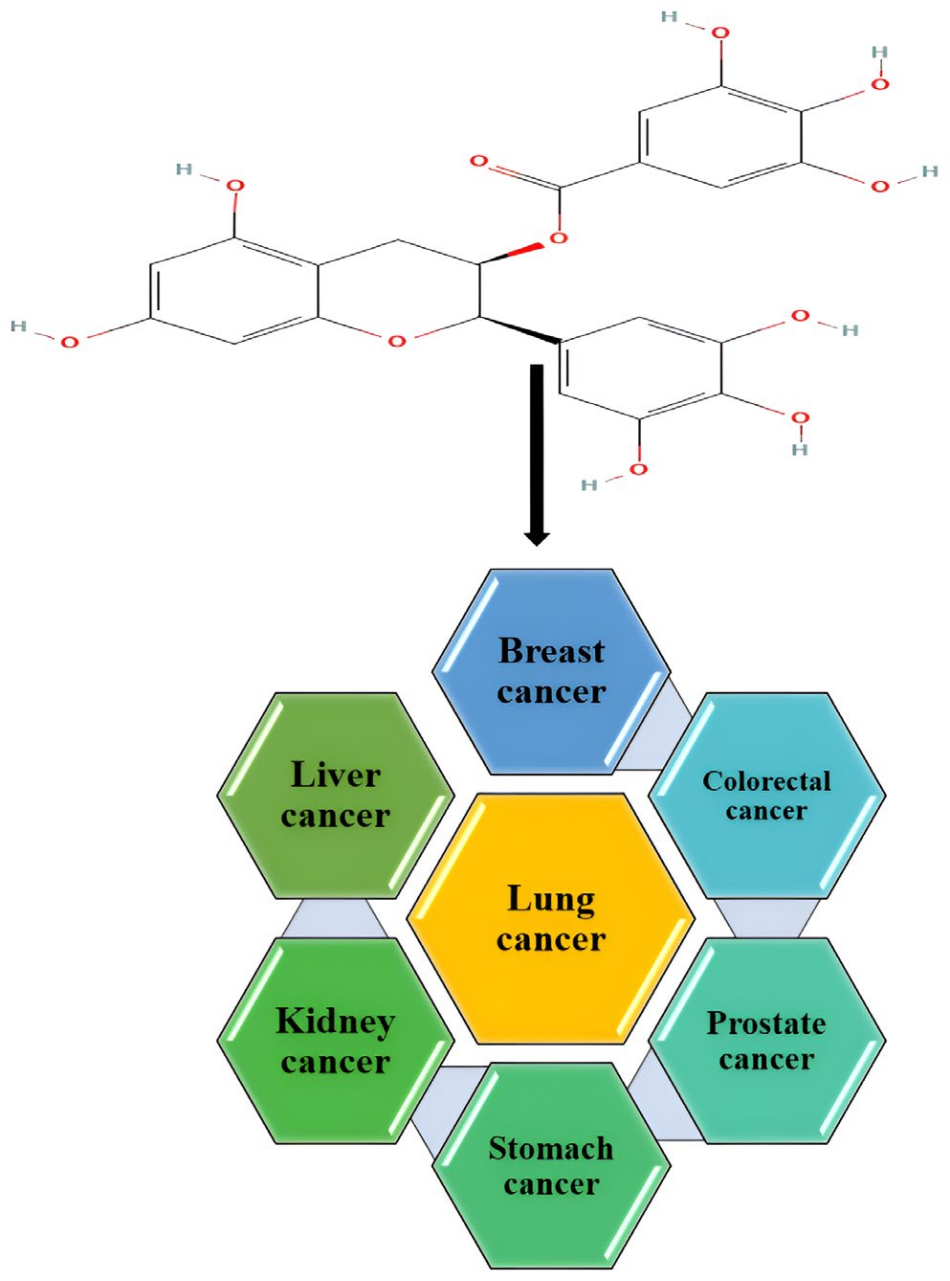
Anticancer perspectives of EGCG.

**FIGURE 2:**
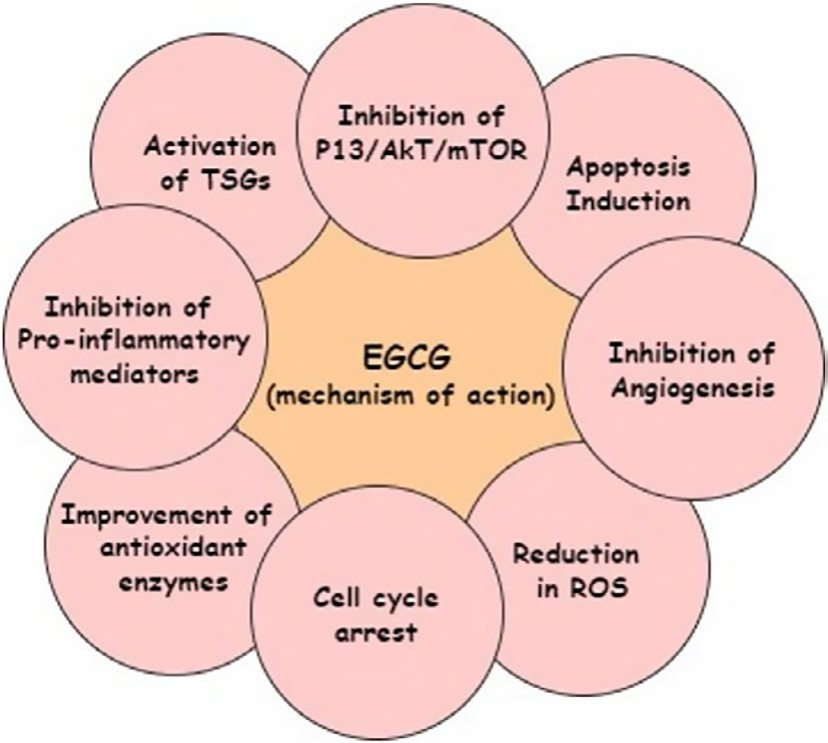
Anticancer mechanisms of EGCG.

The developing countries are facing rapid cancer progression, and prevalence is increasing every day. Numbers of factors, including poor and unhygienic dietary intake, polluted environmental settings, and pathogenic diseases, are contributing significantly to oncogenesis. Oxidative stress, inflammation, DNA damage, and genetic mutations are such events that occur in cancer incidence. However, the chronic condition can be controlled and minimized through proper dietary strategies and an integrative approach. Plant‐based bioactive compounds are natural compounds that have the potential to decrease the risk factors of cancer development. The EGCG is a flavonoid belonging to polyphenols, mainly found in green tea and grapes, and has been reported with anticancer potential. Figure 1 presents the anticancer perspectives of EGCG, and Figure 2 shows the anticancer mechanisms of EGCG.

### 5.1 Lung Cancer

Lung cancer (LC) is the leading cancer among all cancers, encompassing small cell lung cancer (SCLC) and non‐small cell lung cancer (NSCLC). The morbidities and mortalities from LC in 2024 will be estimated to reach ~234,580 and ~125,070 respectively. A number of factors are involved in LC, and smoking is the main risk factor responsible for ~85% of cases; however, occupational chemical and heavy metal exposure, family history, and air pollution are also linked with lung cancer (Thandra et al. 2021). Smoking can lead to dysplasia of the lung epithelium, and upon continuation, it may cause genetic alterations and affect protein synthesis. Genetic mutations in MYC, BCL2, and p53 genes are responsible for causing SCLC, and NSCLC occurs due to p16, EGFR, and KRAS gene mutations (Lindeman et al. 2018). Moreover, the dysfunction of the RB family (p107, p130), tumor suppressor PTEN, chromatin regulator CREBBP, and NOTCH receptors are other factors contributing to cancer pathogenesis. The deletion of the 3p14–23 chromosome is a crucial chromosomal modification specifically associated with SCLC, and the induction of the PI3K/AKT/mTOR pathway has been related to SCLC progression (Lázaro et al. 2022; Kern et al. 2020). Investigations have proved that a possible biochemical way to reduce the LC burden is via downregulation of p21, pAkt, Akt, and MMP‐2 protein expression, reduction of BCL2 and BCLXL mRNA levels, whereas improved BAX mRNA expression, RAD50 protein levels, and CASPASE3 and caspase‐3 activity in LC cells (Baruah et al. 2020).

**FIGURE 3:**
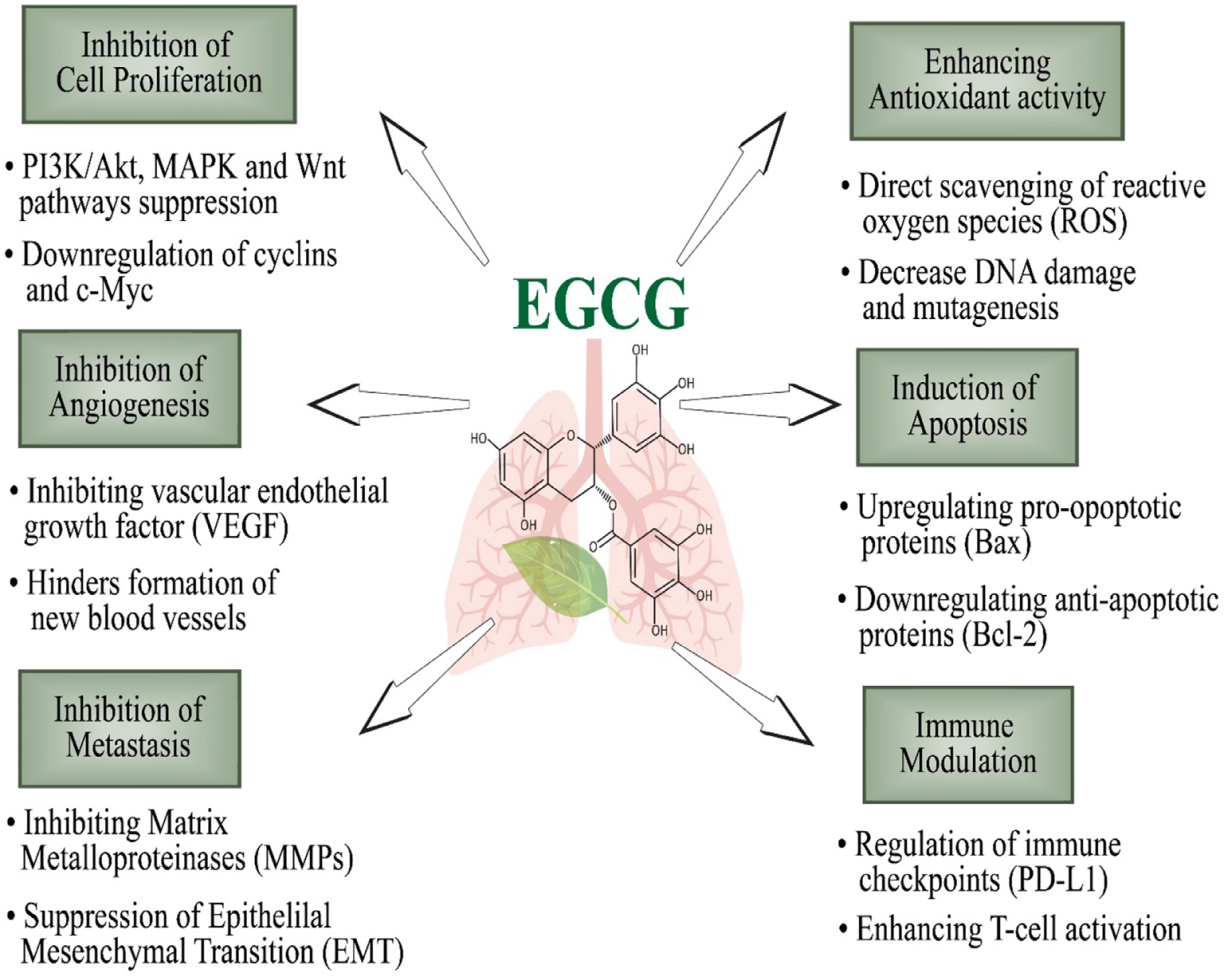
Anticancer activity of EGCG against lung cancer via immune modulation, apoptosis induction, inhibition of metastasis, angiogenesis, and cell proliferation, improved antioxidant capacity, and downregulating MMPs, VEGF, and Wnt/P13K/Akt axis.

The study of Jiang et al. (2018) proved the anticancer effect of EGCG (20 mg/kg) in LC stem cells of mice via modulating the hsa‐mir‐485‐5p/RXRα axis and triggered cell apoptosis in stem cells. Recently, Moar et al. (2024) explored in vitro anticancer potential of EGCG on A549 NSCLC cell lines through MTT assay. The study concluded that EGCG (50 μM) reduced A549 cell proliferation and migration. EGCG combined with tyrosine kinase inhibitors (TKIs) efficiently stimulated the AMPK pathway, suppressed the ERK/MAPK and AKT/mTOR pathways, induced apoptosis, and cell cycle arrest in drug‐resistant NSCLC cells (Zhou et al. 2023). Minnelli et al. (2021) studied the EGCG anticancer effect in NSCLC cell lines via targeting EGFR and reported declined cell proliferation and enhanced apoptosis in cancer cells. Namiki et al. (2020) reported that EGCG and GTE activated the AXL receptor tyrosine kinase, inhibited SLUG, p‐AXL, and ALDH1A1 in H1299‐sdCSCs in LC stem cells. Hu et al. (2019) investigated EGCG anticancer activity on lncRNAs and mRNAs in LC and concluded cell cycle arrest and reduced cell proliferation. Bhardwaj and Mandal (2019) studied the EGCG role in modulation of A549 cell microRNAs using next‐generation sequencing and reported MAPK pathway regulation and G0/G1 phase arrest. Gu et al. (2018) reported EGCG induced apoptosis in LC cells via preventing the PI3K/Akt signaling pathway. Previously, Jing‐Jing et al. (2013) highlighted the EGCG role in LC A549 lines through Bcl‐xl expression downregulation, Bax mRNA and protein expression upregulation, and Ku70 expression inhibition. Figure 3 showed the anticancer activity of EGCG against lung cancer via immune modulation, apoptosis induction, inhibition of metastasis, angiogenesis, and cell proliferation, improved antioxidant capacity, and downregulating MMPs, VEGF, and the Wnt/P13K/Akt axis.

### 5.2 Pancreatic Cancer

Pancreas is performing double function as exocrine and endocrine gland, releasing insulin hormone, which controls and regulates blood glucose level. Insulin and other endocrine hormones including glucagon, somatostatin, proinsulin, pancreatic polypeptide (PP), amylin, and C‐peptide are produced from Langerhans islets (Karpińska and Czauderna 2022). Pancreatic carcinoma is the 4th leading cause of cancer mortalities in the US and may cross colorectal cancer (CRC) before 2040, mostly occurring from pancreatic duct cells, referred to as pancreatic ductal carcinoma (PDC). The etiological studies proved that smoking, age, diabetes, obesity, *H. pylori* infection, chemical exposure, liver cirrhosis, and chronic pancreatitis are potential risk factors of carcinoma (Hu et al. 2021). Chronic pancreatitis (CP) is frequently connected with parenchymal fibrosis, permanent lesions within exocrine and endocrine pancreatic tissue, ultimately leading to PDC. The CP is involved in genetic mutation and the genes (PRSS1, FTR, SPINK1, TRPV6, and CTRC) that once get mutated may lead to PDC. Likewise, CP is linked with amendment of molecular mechanisms like TGF‐β signaling, Wnt/notch signaling, G1/S checkpoint, and KRAS signaling, and the modification of KRAS oncogene on codon 12 is the starting event in several PDC cases, followed by the inactivation of INK4a, TP53, and DPC4 TSGs (Saiki et al. 2021).

Hypoxia‐inducible factor‐1 (HIF‐1), a transcription factor, is often involved in cancer pathogenesis. Hu, Xu, et al. (2023); Hu, Yang, et al. (2023) studied invitro and invivo anticancer effects of EGCG on MiaPaCa‐2 and PANC‐1 pancreatic cell lines via targeting HIF‐1. They reported EGCG reduced tumor‐induced HIF‐1α by inhibiting cancer‐induced insulin receptor (IR) and IGF1R. It has been reported that histone deacetylase inhibitors (HDACIs) are anticancer agents that induce apoptosis and cycle arrest via mitochondrial and death receptor pathways. Sanaei et al. (2022) explored the anticancer effects of sodium butyrate and EGCG in CAPAN‐1, PA‐TU‐8902, and CFPAC‐1 pancreatic cell lines through BAK, APAF1, BAX, Bcl‐2, Bcl‐xL, p21, and p53 expression. The study verified that both therapeutic agents effectively altered gene expressions and stopped cell proliferation. Wei et al. (2019) reported invivo and invitro inhibition of pancreatic cell migration via reduced TCF8/ZEB1 and β‐Catenin expression, suppressing IGFR phosphorylation and Akt dysregulation, upon treatment with EGCG. Lu et al. (2019) reported a synergistic anticancer effect of EGCG, chlorogenic acid, and thermal cycling‐hyperthermia against pancreatic PANC‐1 cells by cell cycle arrest at the G2/M phase and initiation of ROS‐dependent mitochondria‐mediated cell death. Earlier, Shankar et al. (2013) described that EGCG treatment inhibited pancreatic cancer in Balb C nude mice through downregulating AKT, ERK, PI3K, and FKHRL1/FOXO3a, and modulating FOXO genes. Moreover, EGCG regulated caspase‐3, p27/KIP1, CD31, VEGF, HIF1α, MMP2, MMP7 and upregulated N‐cadherin and Zeb1 expression. Shortly, EGCG showed anticancer potential via inhibiting PI3K/AKT and ERK pathways and stimulation of FKHRL1/FOXO3a.

### 5.3 Breast Cancer

Breast cancer (BC) ranked 2nd in different types of cancers with respect to prevalence worldwide, and according to WHO, ~2.3 million women were identified with BC, and ~670,000 deaths were reported due to BC in 2022 (Nardin et al. 2020). Numerous risk factors, including gender, hormonal changes, age, physiological conditions, dietary, and lifestyle behavior are accounted for in BC; however, hormonal imbalance and fluctuations have a notable contribution to cancer development. High levels and lengthy exposure to estrogen, an important hormone linked with augmented risk of cancer progression, are significant (Dall and Britt 2017). Postmenopausal females with high estrogen levels, early menstruation (~12 years) and delay in termination (~50 years) are most susceptible to developing BC, compared to females who started menstruation late (~15 years) and completed it early (~40 years). Additionally, hormonal replacement therapy (HRT) is also a key risk factor for BC; however, the risk can be limited if HRT is used after 60 years (Cohain et al. 2017). Genetic mutations are only counted in 5%–10% of cases of BC development, and BRCA1 and BRCA2 are important genes associated with cancer progression. BRCA1 and BRCA2 genes, positioned on chromosomes 17 and 13 respectively, are suppressor genes tangled in genomic stability, encrypting nuclear protein and repairing double DNA strand breaks. Some other genes, like CHEK2, ATM, PALB2, and BRIP1, show a modest tendency for BC; however, patients with these genes' mutations have 2–3 times the high risk of developing a malignant tumor (Chamseddine et al. 2022).

**FIGURE 4:**
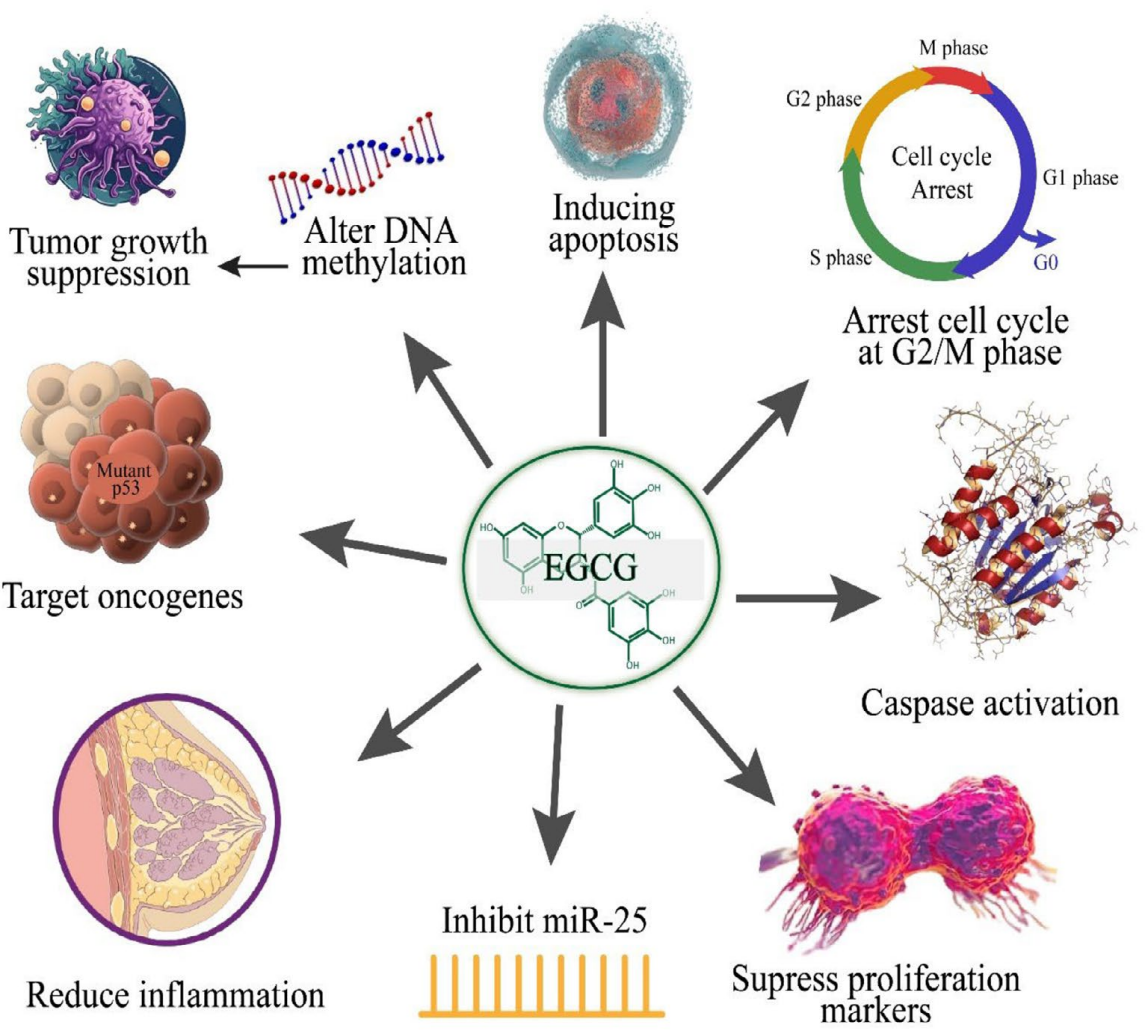
Anticancer activity of EGCG against BC via miR‐25 inhibition, apoptosis induction, caspase activation, proliferation markers reduction, cell cycle arrest, tumor growth suppression, and regulation of oncogenes.

Studies on EGCG on BC proved its effectiveness by highlighting multiple apoptotic pathways, markers, and mediators. Nag et al. (2024) investigated EGCG‐loaded lipid‐based NPs against BC cells and concluded PI3K/Akt/mTOR pathway dysregulation and reduced angiogenesis through inhibiting VEGF. EGCG has been stated to induce anticancer activity by reducing proliferation, enhancing apoptosis, inhibiting inflammation, and DNA demethylation (Marín et al. 2023). Mutation in p53, which is generally recognized as an oncogene, induces many oncogenic activities. Considering this, Kollareddy and Martinez (2021) evaluated EGCG effects on mutant p53 protein in triple‐negative breast cancer cells. The findings proved the reduction of ETS transcription family members i.e., ETV1, ETS1, ETV4, and ETS2, which are tangled in cell metastasis. In addition, decreased protein levels of p53 and reduced expression of GMPS and IMPDH1 targets promote invasion. EGCG delivery via NPs is an innovative strategy to improve efficiency in breast cancers. Radhakrishnan et al. (2019) developed bombesin‐EGCG conjugated solid lipid NPs to evaluate in vitro and in vivo efficacy against BC. They concluded that reduced tumor volume and augmented survival time for tumor‐bearing mice. Zan et al. (2019) assessed EGCG anticancer activity in MCF‐7 cells by targeting miR‐25. They resulted in a decline in miR‐25 expression, augmented PARP, caspase‐3/9 at the protein level, interrupted the cell cycle at the G2/M phase, and triggered apoptosis. Furthermore, miR‐25 and Ki‐67 expression also reduced in an in vivo analysis. Wei et al. (2018) reported the anticancer potential of EGCG (5, 10, 15 mg/kg/day) in Balb/c mice via reducing breast tumor weight and VEGF. Figure 4 showed the anticancer activity of EGCG against BC via miR‐25 inhibition, apoptosis induction, caspase activation, proliferation markers reduction, cell cycle arrest, tumor growth suppression, and regulation of oncogenes.

### 5.4 Prostate Cancer

Prostate cancer (PC) is the 2nd most prevalent cancer among men; the prostate gland, present below bladder, produces and forces semen through the urethra upon ejaculation and gets larger with age. The common prostate gland problems are prostate cancer, prostatitis, and benign prostatic hyperplasia (BPH). According to ACS, ~299,010 new cases and ~35,250 deaths from prostate cancer have been reported, which means 1 in 8 men can be identified with prostate cancer during their lifetime (Gandaglia et al. 2021). Various processes, including prostatic intraepithelial neoplasia (PIN) and androgenic regulation of prostate cancer, are fundamental processes responsible for PC progression. Androgenic regulators (ARs) are critical transcription factors in cancer development through the nuclear translocation of receptors, triggering the transcription of genes, proliferation, cell differentiation, and apoptosis (Dahiya and Bagchi 2022). Several growth factors like TGF‐β, IGF, EGF, and FGF are ARs dependent, and EGF, with its membrane‐related tyrosine receptor kinase EGF‐1, is responsible for the progression of cancer cells by enhancing migration. Moreover, TSG like PTEN negatively modulates the PI3K/AKT/mTOR pathway and stumbles cell‐cycle at the G1 stage, hence causing cell proliferation. Thus, the dysfunction of PTEN results in an upsurge of the PI3K/AKT/mTOR pathway and blights normal AR regulation, subsequently amplifying proliferation and declining apoptosis (Imada et al. 2021). Serine/threonine (PIM‐1) a proto‐oncogene, is vital in cancer development and cell proliferation, and studies have proved that PIM‐1 kinase, involved in cellular development, immunoregulation, and oncogenesis, is an appropriate therapeutic target for PC (Wang, Li, et al. 2017; Wang, Man, et al. 2017).

Suppression of androgenic receptor and apoptotic signaling pathway initiation is a possible and potent strategy to mitigate PC cell migration and invasion. Mohammadi et al. (2024) researched prostate cancer LNCaP cells to evaluate the therapeutic potential of EGCG. They reported that 400 μM EGCG improved apoptotic factors (BAX, CASP3, CASP7) expression, while reducing expression of AR and PSA. Alserihi et al. (2023) inspected the comparative anticancer potential of EGCG and EGCG‐NPs in PC3 and 22Rv1 prostate cell lines. The study proved that both treatments significantly showed anticancer properties. Previously, Alserihi et al. (2021) developed EGCG‐based folic acid NPs to bind FOLR1 receptors and prostate‐specific membrane antigen (PSMA^+^) in a PC model and reported the anticancer potential of EGCG‐FA‐NPs. Zhang, Pan, et al. (2020); Zhang, Yu, et al. (2020); Zhang, Xu, et al. (2020) proved that EGCG reduced PC cell growth by enhancing miR‐520a‐3p and reducing AKT1 expression. Marchetti et al. (2020) stated that EGCG triggers Ca^2+^‐induced Ca^2+^ release through ryanodine and IP3 receptors and thus induces a cytotoxic effect in DU145 and PC3 cell lines. Mokhtari et al. (2020) studied the anticancer impact of EGCG via targeting expression of miRNA. They reported that EGCG treatment modulated miR‐34a mimic and miR‐93; thus, it reduced AR and PSA expression. Yeo et al. (2020) mentioned EGCG anticancer activity in PC3 cell lines through inhibiting the twist/VE‐cadherin/AKT mechanism. Harper et al. (2007) concluded that EGCG suppressed early‐stage PC by reducing AR, IGF‐1, GF‐1R, phospho‐ERKs 1 and 2, COX‐2, and iNOS in rodents.

### 5.5 Liver Cancer

Liver is a main organ responsible for metabolism, detoxification of xenobiotics, synthesis of several proteins, enzymes, and hormones. Lobule is the functional unit of liver, which is hexagonal in shape and composed of hepatocytes. The basic function of liver is production of bile, a fluid that contains various other components involved in digestion and absorption of lipids (Yang 2021). The cytochrome enzyme family is mainly involved in the metabolism and detoxification mechanism. The fat‐soluble vitamins arrive at liver through intestinal absorption in the form of chylomicrons and VLDL, whereas drugs and other xenobiotics undergo hydrophilic form from lipophilic nature in two phases. The phase I is the formation of hydrophilic solute via oxidation, reduction, and hydrolysis by the CYP450 family of enzymes. The phase II includes conjugation of metabolites formed in phase I to convert them into hydrophilic nature for secretion into blood and bile (Almazroo et al. 2017).

Liver cancer or hepatocellular carcinoma (HCC) is affecting ~41,630 people and ~29,840 people will be going to die in 2024 due to HCC, thus making it the 6th common cancer globally. Liver cirrhosis, fatty liver disease (FLD), alcohol consumption, exposure to toxins and chemicals, hepatitis, and congenital disorders are major risk factors related to HCC (Huang et al. 2021). Viral hepatitis, a significant risk factor of HCC, mainly modifies genes such as TERT, PDGFR β, and MAPK1. Moreover, the virus alters other proteins like HBx, which can mutate Ras, JNK, Raf, MAPK, and ERK gene expressions (Wang, Pan, et al. 2021; Wang, Yeh, and Chen 2021). Alcohol produces pro‐inflammatory mediators via monocyte activation and results in augmented circulating endotoxin concentrations, triggering Küpffer cells which release several chemokines & cytokines including IL6, TNFα, IL1β, and prostaglandin E2 (Hillmer et al. 2020). The toxins, specifically aflatoxin and other health hazard chemicals, are also linked with gene mutation, especially TSG p53 expression. The other epigenetic modifications induce dysregulation of TSGs and oncogenes i.e., β‐catenin, MET, ErbB, p16(INK4a), COX2, HGF, and E‐cadherin (Cao et al. 2022).

Currently, Yang et al. (2024) elaborated on the in vitro therapeutic potential of EGCG in HCC and found that EGCG modulated PIK3CA expression, inhibited PI3K/AKT, and reduced proliferation in HepG2 cells. Rodponthukwaji et al. (2023) developed EGCG‐loaded PLGA –siRNA‐based NPs to emulate their potential against HCC cells. They reported that NPs augmented caspase‐3/7 activity and reduced cell growth. Tang et al. (2020) reported reduced cell growth and invasion against hepatic cancer in rats by suppressing cell division cycle 25A. Moreover, EGCG treatment enhanced p21waf1/Cip1 expression in HepG2 and downregulated CDC25A in Huh7 cells. Liao et al. (2020) examined the effectiveness of EGCG‐derived Y_6_ against HCC and concluded that the EGCG derivative Y_6_ significantly reduced angiogenesis and tumor progression via modulating the MAPK/ERK1/2 and PI3K/AKT/HIF‐1α/VEGF axis. Table 3 presents the EGCG anticancer potential against HCC via possible intervention and pathway.

**TABLE 3:** EGCG anticancer potential against HCC via possible intervention and pathway.

	Intervention	In vivo/in vitro	Results	References
Hepatocellular carcinoma (HCC)	EGCG	Hep3B	**↓**ERα36, PI3K/Akt, MAPK/ERK	Chen et al. (2019)
	EGCG	Wistar rats	**↓**Fibrosis, tumor progress	Sojoodi et al. (2020)
	EGCG‐AuNPs	HepG2	**↓**c‐Myc protein, **↑** let‐7a, miR‐34a, caspase‐3	Mostafa et al. (2020)
	EGCG with metformin	HepG2	**↓**cyclin D1, lncRNA‐AF085935, glypican‐3, **↑**caspase3	Sabry et al. (2019)
	EGCG	HepG2	**↓**AFP secretion	Zhao et al. (2017)
	EGCG derivative Y_6_	BEL‐7404/DOX	**↓**HIF1α, cell proliferation, **↑**apoptosis	Wen et al. (2017)
	EGCG	Hep3B, HepG2 and Huh‐7	**↓**PFK activity, **↑**apoptosis	Li et al. (2016)
	EGCG	HCCLM6	**↓**Bcl‐2, NF‐κB, MMP, **↑**Bax, p53, caspase 3/9, cell cycle arrest	Zhang et al. (2015)
	EGCG	HepG2, Sprague–Dawley rats	**↓**α‐fetoprotein, MMP‐9, syndecan‐1, FGF‐2	Darweish et al. (2014)
	EGCG	HCCLM6	**↓**MMP 2/9, metastasis, **↓**apoptosis	Zhang et al. (2013)
	EGCG	HepG2	**↑**ROS, LMP	Zhang et al. (2012)

### 5.6 Kidney Cancer

Kidney cancer is the 10th most common cancer in both genders, accounting for ~4%–5% of cases. It has been estimated that in 2024, ~1610 new cases of kidney cancer will be diagnosed, and ~14,390 people will die from this disease. Kidneys are involved in metabolism, filtration, absorption, and excretion of metabolites, as well as production of hormones and enzymes. Despite its prime significance, the peril of renal cancer has been alarming the global population. The renal cell carcinoma (RCC) is the most common type of renal cancer, and 9 out of 10 renal cancers are RCC. Smoking, obesity/overweight, hypertension (HTN), congenital abnormalities, family history, chronic renal ailments, and exposure to certain chemicals are responsible for cancer development (Scelo and Larose 2018). The pathogenesis of RCC starts from proximal renal tubular epithelium, and structural amendments occur on the short arm of the 3p chromosome. The genes linked with genetic mutations are VHL, BAP‐1, PBRM‐1, SETD2, MTOR, and KDM5C; furthermore, genetic modifications in 5q, 14q, 7q, 8p, and 9p are also responsible for RCC manifestation (Hsieh et al. 2017). The TSG PBRM‐1, which encodes the BAF180 protein, is a major contributor to RCC occurrence. Animal studies have stated its role in cell cycle regulation and replicative senescence; thus, transformed PBRM‐1 results in an abnormal BAF180, which would result in uncontrolled cell growth and consequent tumorigenesis (Liu et al. 2020).

Oxidative stress (OS) plays a major role in gene mutations, thus contributing to cancer progression via altering physiological and biochemical mechanisms. Lyu et al. (2022) studied the anticancer potential of EGCG in renal cancer stem cells (RCSCs) and identified EZH2 as a marker in RCC. The study concluded that EGCG suppressed EZH2, increased apoptosis via modulating KIF11, VEGF, and MMP2 expression, and declined FoxP3+ Treg cells. Chen et al. (2016) reported the downregulation of MMP‐2/9 in RCC, declined invasion and migration when treated with EGCG. Wei et al. (2015) investigated TNF‐related apoptosis‐inducing ligand (TRAIL) as a therapeutic target to manage RCC in 786‐O cell lines. They reported that EGCG and TRAIL suppressed Bcl‐2, c‐FLIP, and Mcl‐1 proteins and induced apoptosis. Sato et al. (2014) described that the Cx32 gene improved vinblastine (VBL)‐induced cytotoxicity and downregulated MDR1 expression. They investigated the EGCG effect on VBL‐induced cytotoxicity in Caki‐1 RCC cell lines. The study highlighted enhanced Cx32 expression in Caki‐1 cells, suppressed MDR1 mRNA expression via inactivation of Src and subsequent activation of c‐Jun NH2‐terminal kinase (JNK).

### 5.7 Bladder Cancer

Bladder cancer affects 1 in 28 men and 1 in 89 women over the age of 55 years or above; thus, its prevalence is common among males rather than females. According to ACS, 4% of cancer cases are bladder cancers in the US, and ~83,190 new cases of bladder cancer and ~16,840 fatalities from bladder cancer will be estimated in 2024. Family history of bladder cancer, genetic mutations, exposure to dyes and chemicals, UTI, and high arsenic content in drinking water are major risk factors for bladder cancer (Alouini 2024). Progression and aggressiveness to penetrate into bladder walls vary according to the type of bladder cancer. The first type is non‐muscle‐invasive bladder cancer, covering ~75% of cases of bladder cancer, and the second is muscle‐invasive bladder cancer, responsible for the rest of the 25% of cases (van Straten et al. 2023). The underlying pathology includes increased RONS, MDA, NO, 8‐iso‐PGF2α, and decreased SOD2 levels in bladder cancer progression. Moreover, genes such as NAT2, TP63, GSTM1, MYC, TACC3‐FGFR3, PSCA, CLPTM1L‐TERT, APOBEC3A‐CBX6, UGT1A, and CCNE1 get mutated in bladder cancer. The overproduction of RONS causes NF‐κB activation, cytokine production, and NOS2 synthesis, leading to ROS/MAPK, ROS/Keap1‐Nrf2‐ARE, and ROS/PI3K/Akt pathways stimulation, linked with bladder cell proliferation (Grębowski et al. 2023).

Urokinase‐type plasminogen activator receptors (uPARs) are glycosylphosphatidylinositol (GPI)‐anchored cell membrane receptors that play a critical role in metastasis and bladder cancer invasion. Sah et al. (2022) assessed the impact of EGCG on uPAR expression in T24 bladder cancer cell lines. They reported that NF‐κB and AP‐1 transcription factors are vital for IL‐1β‐induced uPAR expression and that EGCG treatment suppressed NF‐κB signaling, IL‐1β‐stimulated ROS, ERK1/2, and AP‐1. Yin et al. (2021) studied the anticancer activity of EGCG in 5637 and T24 bladder cancer cells. The results proved that EGCG inhibited proliferation and enhanced apoptosis via improved caspase 3/9, BAX, reduced ATG5, and modulated PI3K/AKT, LC3B II, and Beclin. Sun et al. (2019) stated that EGCG suppressed bladder cancer cells via inhibiting the sonic hedgehog pathway. Feng et al. (2017) mentioned the anticancer activity of EGCG in bladder cancer through TFPI‐2 factor downregulation, inhibited invasion, and induced apoptosis in T24 cells. Liao et al. (2016) investigated the effect of EGCG on T24 bladder cancer cells and reported apoptosis via improved caspase 3/9, BAX expression, and regulated LC3B II, Beclin, and the PI3K/AKT pathway.

### 5.8 Colorectal Cancer

The menace of colorectal cancer (CRC) has been increasing every day, imposing threats to human life. According to estimations ~106,590 new cases of colon cancer and ~46,220 new cases of rectal cancer will be reported, thus making it the 3rd leading cancer and 2nd in casualties globally. The CRC development includes the growth of tiny cell groups (polyps) inside the colon, and somehow with age these polyps transform into tumors in the next 5–10 years. Inherited disorders, genetic alterations, inflammatory bowel illness, and other malignancies are risk factors of CRC progress (Al‐Muswie et al. 2023). Epigenetics and genetics of CRC involved microRNAs (miRNAs) influence cancer‐associated pathways at the post‐transcriptional level, which contribute to CRC progression; metastasis and alterations in oncogenes and TSGs cause dysplastic epithelium in the adenoma–carcinoma process, resulting in CRC development. The CRC pathways include CIN, MSI, and CIMP, and CIC is responsible for ~80%–85% of CRC cases. The CIC is involved in the initiation of growth‐promoting pathways and diminished apoptotic pathways (Fischer et al. 2019). A series of other pathways are also involved in CRC, which are modulated and regulated by oncogenes and TSGs. The APC, a TSG, is usually changed in colorectal cancers, and this mutation stimulates the Wingless/Wnt pathway. This Wnt signaling pathway further transforms KRAS and TP53, leading to the development of polyp cells to cancer, followed by TGF‐β1 mediated cell signaling pathway and accelerated CRC development. The majority of CRC cases are due to altered KRAS and B‐Raf, which activate WNT‐APC‐CTNNB1, PI3K, TGFB1‐SMAD, and RAS–RAF–MAPK pathways and promote proliferation with suppressed apoptosis (Ahronian et al. 2015).

**FIGURE 5:**
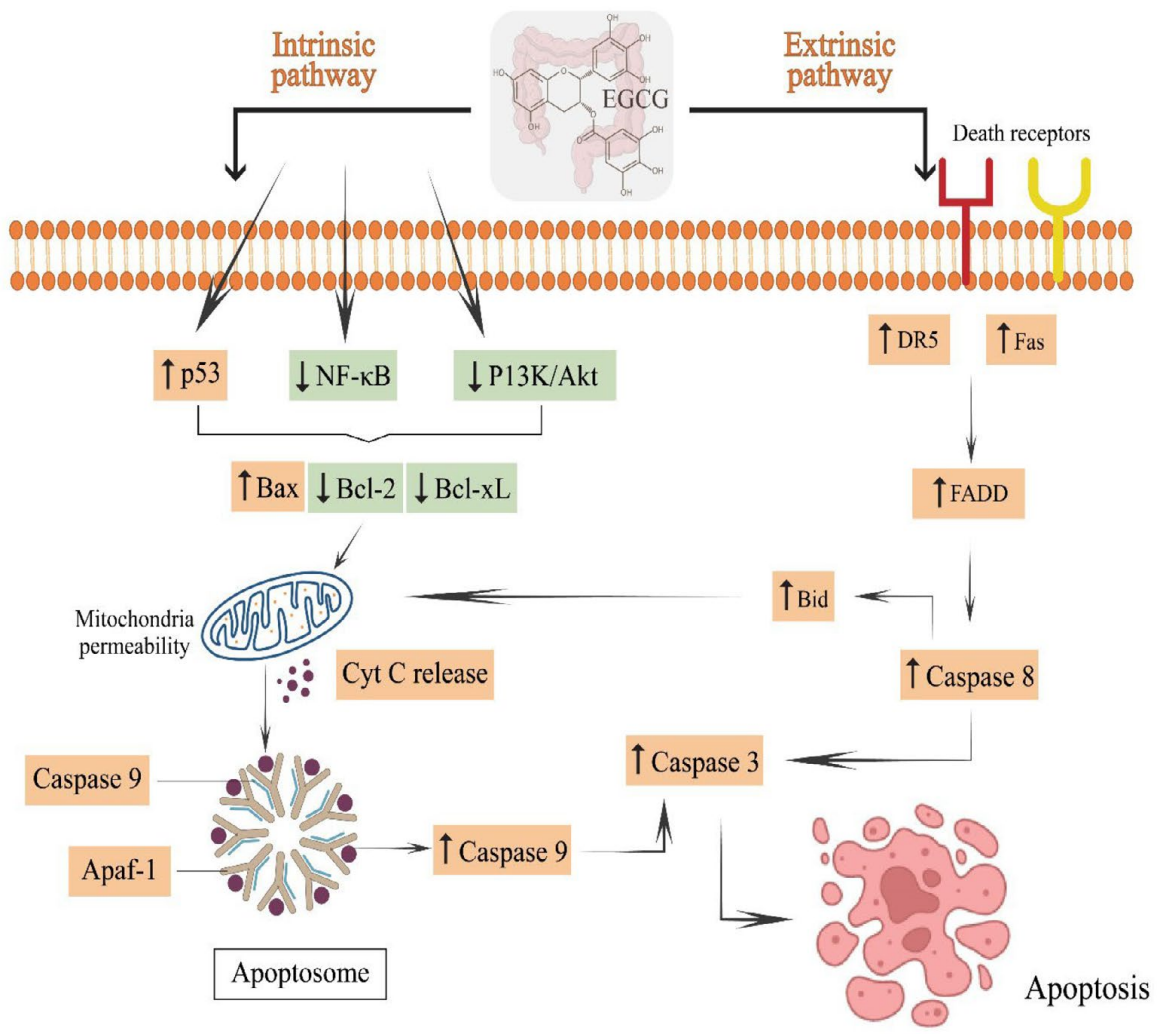
Anticancer activity of EGCG against CRC via P53, BAX, Fas, DR5, caspase 3/8/9 upregulation, apoptosis induction, Bcl‐2, Bcl‐xL suppression, and NF‐κB, P13K/Akt downregulation.

Nano‐delivery of compound/chemical is a suitable and advanced technique to attain desired outcomes. Bhattacharya et al. (2024) formulated pH‐sensitive EGCG NPs to assess anticancer activity against HT‐29 CRC cell lines and verified anticancer potential via enhanced apoptosis and cell cycle arrest. Ibrahim et al. (2024) verified the anticancer effect by cytotoxicity of combined EGCG and 5‐Fluorouracil in CRC stem cells. Chen et al. (2022) studied in vitro EGCG effect on cancer‐associated fibroblasts (CAFs), which have the ability to cause CRC. They found that EGCG suppressed CAFs ability to induce cell proliferation through inhibited glycolytic activity, silencing MCT4, and blocking lactic acid efflux of CAFs. Fatty acid metabolism is a significant hallmark of cancer cells, and EGCG serves as an effective fatty acid and energy metabolism modulator to suppress CRC via regulation of vital genes accounted for fatty acid *de novo* synthesis, AMPK activation, improved FASN expression, and declined ATP production in CRC cells (Wang, Pan, et al. 2021; Wang, Yeh, and Chen 2021). Wang et al. (2020) stated that EGCG (50, 100, 200 mg/kg/day) showed anticancer activity in rats via modulation of various pathways such as p53, PI3K‐Akt, I‐kB kinase/NF‐kB, and apoptotic signaling pathways. La et al. (2019) mentioned that EGCG showed anticancer effect by augmenting the sensitivity of CRC cell lines (HCT‐116, DLD1) to 5‐FU via inhibited GRP78 expression, activated NF‐κB, and improved miR‐155‐5p level. Md Nesran et al. (2019) evaluated the EGCG anticancer effect against CRC through activating endoplasmic reticulum (ER) stress. They found that EGCG reduced CRC progression in HT‐29 cell lines by activating the PERK/p‐eIF2α/ATF4/IRE1α axis and induced apoptosis by caspase 3/7 activity. Figure 5 showed the anticancer activity of EGCG against CRC via P53, BAX, Fas, DR5, caspase 3/8/9 upregulation, apoptosis induction, Bcl‐2, Bcl‐xL suppression, and NF‐κB, P13K/Akt downregulation.

### 5.9 Uterine Cancer

Uterine cancer (UC) is cancer of female reproductive organs, affecting women aged above 60. According to ACS, it has been estimated that ~67,880 new cases of uterine cancer will be diagnosed and ~13,250 women will die from uterine cancers in 2024. Uterine cancer can be categorized as endometrium, developing from the lining of the uterus, which accounted for 95% of cases, and myometrium or uterine sarcomas developing in the muscle tissue, a rare form of uterine cancer (David et al. 2020). Endometrial cancer (EC) is related to several risk factors such as obesity, certain medications, hormonal imbalance, PCOS, age, T2DM, family history, and previous records of breast or ovarian cancer. The genetic mutation is a crucial aspect of EC development, involving the modification of MMR genes (MLH1, MSH2, PMS2 and MSH6). The picture between the BRCA gene family and EC has remained unclear; however, the links with BRCA1 and BRCA2 have been proven by some studies (Long et al. 2019). There is a direct role of augmented levels of circulating E2 in EC development, and it has been verified that E2 and stromal cell‐derived pathways activate the estrogen receptor (ER). Multiple pathways such as SDF‐1alpha/CXCR4 and HGF/c‐Met activate kinases like MAPK and PI3K/AKT, which phosphorylate ER and cause activation of CDKN1A and specific growth factors i.e., IGF‐1, TGF, and MAD2L1, leading to cell proliferation and malignancy (Winuthayanon et al. 2017). Genetic mutations in genes like ARID1A (10%–25%), PTEN (10%–50%), KRAS (10%–15%), TP53 (60%–90%), PPP2R1A (15%–30%), and FBXW (10%–40%) are reported for UC cases, and TP53 and FBXW7 mutations are most frequent in UC (Cuevas et al. 2019).

The study of Man et al. (2020) proved EGCG's anticancer potential against RL95–2 and AN3 CA EC cells through apoptosis induction by modulating ERK/JNK, p38 MAPK, and Akt pathways. Huang et al. (2020) stated the anticancer activity of EGCG against endometrial, breast, and ovarian cancers via Nrf2 activation, NF‐κB downregulation, and interaction with DNMTs and histone deacetylases (HDACs). Wang et al. (2018) described that EGCG inhibited EC development through suppressing the PI3K/AKT/mTOR/HIF1α pathway, inhibiting CXCL12‐induced migration, and reducing angiogenesis. EGCG has the ability to reduce HIF‐1α, VEGFA, and CXCR4 expression in RL95‐2 EC cells; therefore, it subdued cell proliferation and induced apoptosis to suppress EC progression (Wang, Li, et al. 2017; Wang, Man, et al. 2017). Recently, Hung et al. (2024) and Baranowska‐Wójcik et al. (2024) proved that EGCG can inhibit EC through targeting molecular targets (PXK), reducing AKT‐mediated angiogenesis, modulating the EGF/HIF‐1a/VEGF pathway, and synergetic interaction with gut microbiota respectively.

### 5.10 Gastric Cancer

Stomach is associated with food digestion, acts as a reservoir of food, and involves gastrointestinal motility. The stomach/gastric cancer (GC) is affecting 6 of every 10 people above 65, and the prevalence is higher among men (1 in 101) rather than females (1 in 155). According to the statistics, ~26,890 new cases of stomach cancer and ~10,880 deaths from GC will be estimated in 2024. The main root cause of GC is dietary behavior along with lifestyle habits. The *H. pylori* infection, salty foods, obesity, smoking, alcohol, Epstein–Barr virus (EBV), nitroso compounds, low folate intake, and occupational exposures are major risk factors of GC (Thrift and El‐Serag 2020). Former studies have affirmed the direct association of *H. pylori* with gastric carcinoma, and a suitable environment with an impaired immune system of the host can increase the risk of cancer incidence. This gram‐negative bacteria secretes multiple substances like urease, acetaldehyde, protease, phospholipase, and ammonia, subsequently resulting in mucosal damage through urease‐mediated myosin II stimulation (Dincă et al. 2022). The *H. pylori* is involved in the production of RONS and OS leading to DNA damage via NF‐κB and Wnt/β‐catenin activation. Epidemiologic evidence advocates that *H. pylori* strains comprising cag pathogenicity island (cagPAI) are more contagious because cagPAI is a 40‐kb genome segment that encodes ~30 genes, including cytotoxin‐linked gene A (cagA). Consequently, *H. pylori* acts through cagA to stimulate growth factor receptors, upsurge proliferation, prevent apoptosis, and enhance angiogenesis (Zhang et al. 2022).

The dietary behavior such as excess salt and salty foods has been reported in gastric cancer development. Saturated NaCl (S‐NaCl) encourages the development of N‐methyl‐N′‐nitro‐N‐nitrosoguanidine induced gastric carcinomas (Balendra et al. 2023). Overexpression of the cell surface receptor c‐erbB2 of the tyrosine kinase family, altered K‐ras oncogene, and irregularities in the FGFR2/ErbB3/PI3 kinase pathway have been widely related to GC (Zhu 2020). The dysfunction of TSG p53 and RUNX3 characterized by GC‐AT transitions, caused by carcinogenic N‐nitrosamines and *PTEN* inactivation on chromosome 10q23.31 are major contributors to carcinoma (Song et al. 2020). Alterations in several growth factors involved in GC progression further produce mediators that worsen the conditions. The increased expression of TGFBR2, CDC25A, SMAD7, and RELA and downregulation of p27 are major events in cell proliferation and cancer invasion (Kumari et al. 2021).

Recently, Shahriari Felordi et al. (2023) targeted the c‐FLIP/Ku70 complex in MKN‐45 GC cells to evaluate EGCG anticancer activity. They reported that EGCG triggered apoptosis in MKN‐45 cells by upregulating P53 and P21, downregulating c‐Myc and Cyclin D1, and inducing cycle arrest in G2/M phases. Furthermore, EGCG suppressed the c‐FLIP and Ku70 expression and reduced their interaction. Xue et al. (2021) evaluated the combined effect of EGCG (25 μg/mL) and cisplatin (5 μg/mL) against BGC‐823 GC cells. The combined therapy induced cell death through the activation of p19^Arf^‐p53‐p21Cip1 signaling pathway and initiated cell cycle arrest in the G1 phase. Zhao et al. (2020) reported EGCG anti‐tumor effects in GC by reducing cell proliferation and migration via modulation of long non‐coding RNA, LINC00511, and the miR‐29b/KDM2A axis. Milani et al. (2019) illuminated the cytotoxic potential of EGCG and trans‐cinnamaldehyde (TC) against AGS cells. The combined therapy induced apoptosis and reduced cell proliferation, thus proving the cytotoxicity of EGCG. Table 4 explained the EGCG anticancer potential against GC via possible intervention and pathway.

**TABLE 4:** EGCG anticancer potential against GC via possible intervention and pathway.

	Intervention	In vivo/in vitro	Results	References
Gastric cancer (GC)	EGCG loaded hyaluronic acid and doxorubicin NPs	MKN45	**↓**Cell proliferation, gastric tumor activity, **↑**Cell cycle arrest, apoptosis	Mi et al. (2018)
	FU‐CMC‐EGCG‐GNPs	Mice infused with MKN45	**↓**Progression of gastric tumor cells	Yuan et al. (2018)
	FU‐CMC‐EGCG‐GNPs	Invivo	**↓**Cell proliferation	Yuan et al. (2018)
	EGCG	SGC‐7901	**↓**Wnt/β‐catenin signaling	Yang et al. (2016)
	EGCG with 5‐FU	SGC‐7901/5‐FU	**↓**Bcl‐2, ABCG2, P‐gp, MDR‐1, GST‐π, **↑**PARP, Bax	Tang et al. (2016)
	EGCG	SGC‐7901	**↓**Wnt/β‐catenin signaling, **↑**apoptosis	Yang et al. (2016)

### 5.11 Bone Cancer

Bone cancer is affecting almost every age of people, accounting for less than 1% of all cancers. According to ACS, ~3970 new cases have been diagnosed and ~2050 deaths will be estimated from bone cancer in 2024. Osteosarcoma (OC) is the most common type of bone cancer, followed by chondrosarcoma and Ewing tumors/Ewing sarcoma (Hu, Xu, et al. 2023; Hu, Yang, et al. 2023). Multiple risk factors such as age, hormones, diet, alcohol, obesity, sunlight, pathogens, inflammation, chemicals, and toxins are contributing to bone cancer prevalence. Osteosarcoma is credited to chromosomal aberration and changes in p53, Rb1, and deoxyribonucleic acid repair genes, while chondrosarcoma is linked with EXT1/2, TP53, Rb1, and IDH1/2 gene alterations. The last Ewing sarcoma is due to chromosomal translocations, leading to melding an FET protein to an ETS transcription factor, most frequently FLI1(Grünewald et al. 2018).

Osteosarcoma, being the most common bone cancer, has been frequently discussed in the literature, and various factors are involved in the malignancy. The p53 and retinoblastoma (Rb) genes are renowned TSGs, and the dysfunction of these genes is a crucial factor in cancer progression. The 50% of all cancers and 22% of osteosarcomas are due to p53 gene mutation. The p53 gene shows its tumor‐suppressor effects through activating the proapoptotic Bax and p21. The latter deactivates the G1/S‐Cdk and S‐Cdk complexes, leading to cell cycle arrest in G1 (Pan et al. 2021). The transcription factor AP‐1 complex regulates cell division, differentiation, and bone development, which is compromised by Fos and Jun proteins, part of the c‐fos and c‐jun proto‐oncogenes. Jun and Fos are significantly involved in high‐grade osteosarcomas and metastasis development (Lv et al. 2019). The overexpression of Myc, another transcription factor that is involved in cell division stimulation, has been reported in OC, and the downregulation of Myc results in cell cycle arrest. Likewise, growth factors like TGF, CTGF, IGF, and TGF‐β play a crucial role in bone matrix synthesis, proliferation, and apoptosis. Furthermore, bone morphogenic proteins (BMPs) are a major part of the TGF‐β family and are associated with high‐grade osteosarcomas (Xie et al. 2023).

Currently, Ren et al. (2023) investigated EGCG‐based nano‐hydroxyapatite against OC and reported that HA‐EGCG NPs exhibited greater anti‐osteosarcoma effects in vitro and in vivo. Dong et al. (2022) reported EGCG in vitro anticancer potential against osteosarcoma by inhibition of the Wnt/β‐catenin signaling pathway. Bolivar et al. (2018) reported that EGCG effectively inhibited UMR 106–01 BSP cells in OC and downregulated CXCR‐4, VEGF‐A, MYC, and PD‐L1 expression in OC cells. Dong ChaoQun et al. (2017) investigated EGCG inhibitory effect in the 143B OC cell line and concluded that 143B cell migration was significantly inhibited, upregulated caspase‐3 expression, downregulated Bcl‐2, MMP‐2, β‐catenin, c‐Myc, and cyclin D1 protein expressions, thus reducing OC invasion via modulating the Wnt/β‐catenin signaling pathway. Tang et al. (2015) reported EGCG tumor inhibitory effect in OC cells through suppressing the MEK/ERK signaling pathway. Jiang et al. (2014) described EGCG anticancer activity in MG63 and U2OS OC cells via increased miR‐126 expression induced apoptosis.

### 5.12 Cervical Cancer

Cervical cancer (CC) is the 4th most common cancer among women, with ~660,000 new cases and about 350,000 expiries reported in 2022. According to estimation, ~13,820 new cases of invasive CC will be diagnosed and ~4360 women will perish from CC in 2024. The frequency is high among women with an age of 35–44 and low in younger females. Smoking, impaired immunity, multiple sex partners, and sexually transmitted pathogens are risk factors for cervical cancer. However, human papillomavirus (HPV) is known for more than 75% of CC cases (Zhang, Pan, et al. 2020; Zhang, Yu, et al. 2020; Zhang, Xu, et al. 2020). Among various strains of HPV, types 16 and 18 are the most notorious due to causing high‐grade CC. The onco‐proteins (E6, E7) of HPV disrupt the host cell cycle, especially E6, which disturbs tumor suppressive protein (p53) and apoptosis signaling cascade proteins, like Bak, FADD, and procaspase 8, while E7 interacts with retinoblastoma protein (pRB). Alongside, E5 protein may play a role in immune dysfunction and oxidative stress, and microRNAs also play a role in cervical carcinogenesis (Romero‐Masters et al. 2022).

The studies on EGCG potential against CC highlighted its therapeutic effect by regulating genetics and epigenetics. It has been reported that EGCG nano‐formulations proved efficient in CC therapy via regulating genes, MicroRNA targets, and DNA methylation patterns (Wang et al. 2023). Panji et al. (2021) studied EGCG (0–100 μmol/L) anticancer effect in HeLa and SiHa CC cell lines via targeting TGF‐β‐induced EMT process. The results showed that EGCG inhibited TGF‐β impact, improved E‐cadherin expression, and reduced phosphorylation of Smad2/3. McDonnell et al. (2019) illuminated the combined anticancer effect of EGCG and Enoxacin in CC cells through reduced cell proliferation and apoptosis. Likewise, Pal et al. (2019) also verified the combined anticancer potential of EGCG and eugenol against CC cells via downregulating cyclinD1 and upregulating cell cycle inhibitors (LIMD1, RBSP3, p16) at the G1/S phase. Hussain (2017) highlighted EGCG's effective role in HeLa cell lines through improved GPx and SOD activity. Kilic et al. (2015) studied the combined anticancer potential of EGCG and cisplatin in CC HeLa cell lines and concluded that the combined strategy modulated p‐mTOR, NFκB/p65, and COX‐2, p‐Akt pathways, while improving Nrf2/HO‐1 expression. Khan et al. (2015) reported EGCG's anticancer effect in CC via modulating TSGs by suppressing DNMT and HDAC expression.

## 6 Conclusion

EGCG, an ester of EGC and gallic acid, present in green tea, is a significant polyphenolic bioactive compound. Absorption and bioavailability of EGCG are extremely poor; however, methylation of EGCG, substitution of the hydroxyl group in EGCG, and addition of acylated long‐chain fatty acids are effective ways to enhance its absorption and bioavailability. Moreover, gut microbiota and nano‐delivery can also significantly contribute to improved bioavailability. The rich antioxidant capacity enables it to combat RONS and reduce tumorigenesis. Numerous anticancer studies, including clinical trials, have proved its efficiency in cancer management. The anticancer‐based studies proved its effectiveness against different cancer types, i.e., lung cancer, breast cancer, bone cancer, liver cancer, bladder cancer, renal cancer, and colorectal cancer. The possible mechanisms of anticancer activity involved activation of TSGs, improved antioxidant enzyme systems (GPx, SOD, GSH), cell proliferation inhibition, reduced angiogenesis, cell cycle arrest, and induction of apoptosis. Moreover, anticancer pathways modulation, pro‐inflammatory markers' (IL‐6, TNF‐α) suppression, and downregulation of oncogenes are other potent processes. Combining all of the above EGCG is a remarkable bioactive component with high antioxidant and anticancer potential.

## Author Contributions

**Ahmad Mujtaba Noman:** conceptualization (equal), writing – original draft (equal). **Muhammad Tauseef Sultan:** conceptualization (equal), writing – original draft (equal). **Aimen Mazhar:** data curation (equal), writing – review and editing (equal). **Iqra Baig:** investigation (equal), writing – original draft (equal). **Jawaria Javaid:** investigation (equal), writing – review and editing (equal). **Muzzamal Hussain:** investigation (equal), resources (equal), supervision (equal). **Muhammad Imran:** data curation (equal), validation (equal), visualization (equal). **Suliman A. Alsagaby:** investigation (equal), methodology (equal), writing – review and editing (equal). **Waleed Al Abdulmonem:** data curation (equal), investigation (equal), software (equal). **Ahmed Mujtaba:** data curation (equal), validation (equal), visualization (equal). **Tadesse Fenta Yehuala:** data curation (equal), supervision (equal), writing – review and editing (equal). **Mohammed M. Ghoneim:** writing – review and editing (equal). **Ehab M. Mostafa:** data curation (equal), investigation (equal), writing – review and editing (equal). **Mohamed A. Abdelgawad:** data curation (equal), resources (equal), writing – review and editing (equal).

## Conflicts of Interest

The authors declare no conflicts of interest.

## Data Availability

The data that support the findings of this study are available on request from the corresponding author.
